# Automatic Analysis of MRI Images for Early Prediction of Alzheimer’s Disease Stages Based on Hybrid Features of CNN and Handcrafted Features

**DOI:** 10.3390/diagnostics13091654

**Published:** 2023-05-08

**Authors:** Ahmed Khalid, Ebrahim Mohammed Senan, Khalil Al-Wagih, Mamoun Mohammad Ali Al-Azzam, Ziad Mohammad Alkhraisha

**Affiliations:** 1 Computer Department, Applied College, Najran University, Najran 66462, Saudi Arabia; 2 Department of Artificial Intelligence, Faculty of Computer Science and Information Technology, Alrazi University, Sana’a, Yemen

**Keywords:** CNN, FFNN, AD, DWT, LBP, GLCM, fusion features

## Abstract

Alzheimer’s disease (AD) is considered one of the challenges facing health care in the modern century; until now, there has been no effective treatment to cure it, but there are drugs to slow its progression. Therefore, early detection of Alzheimer’s is vital to take needful measures before it develops into brain damage which cannot be treated. Magnetic resonance imaging (MRI) techniques have contributed to the diagnosis and prediction of its progression. MRI images require highly experienced doctors and radiologists, and the analysis of MRI images takes time to analyze each slice. Thus, deep learning techniques play a vital role in analyzing a huge amount of MRI images with high accuracy to detect Alzheimer’s and predict its progression. Because of the similarities in the characteristics of the early stages of Alzheimer’s, this study aimed to extract the features in several methods and integrate the features extracted from more than one method into the same features matrix. This study contributed to the development of three methodologies, each with two systems, with all systems aimed at achieving satisfactory accuracy for the detection of AD and predicting the stages of its progression. The first methodology is by Feed Forward Neural Network (FFNN) with the features of GoogLeNet and DenseNet-121 models separately. The second methodology is by FFNN network with combined features between GoogLeNet and Dense-121 models before and after high-dimensionality reduction of features using the Principal Component Analysis (PCA) algorithm. The third methodology is by FFNN network with combined features between GoogLeNet and Dense-121 models separately and features extracted by Discrete Wavelet Transform (DWT), Local Binary Pattern (LBP) and Gray Level Co-occurrence Matrix (GLCM) methods called handcrafted features. All systems yielded super results in detecting AD and predicting the stages of its progression. With the combined features of the DenseNet-121 and handcrafted, the FFNN achieved an accuracy of 99.7%, sensitivity of 99.64%, AUC of 99.56%, precision of 99.63%, and a specificity of 99.67%.

## 1. Introduction

Alzheimer’s disease (AD) is the most common neurological disease due to a disorder known as cognitive impairment, which progresses to deterioration in cognitive abilities, behavioral changes, and memory loss; AD affects the adaptability that needs to be promoted [1]. Despite the scientific advancement in the medical field, there is no active cure for AD, but the effective method for AD is to slow its progression [2]. Therefore, early detection of Alzheimer’s symptoms in its first stage is vital to prevent its progression to advanced stages [3]. Dementia is one of the most common forms of AD due to the lack of effective treatment for the disease [4]. AD progresses slowly before clinical biomarkers appear. There are changes in the cerebrospinal fluid, such as changes in AB42 by 50% due to increased p-Tau and amyloid deposition in the brain. Thus, the increase in the two states of tau reflects the neurodegeneration causing Alzheimer’s disease. The Mini-Cog is a rapid test that effectively tests memory and evaluates results by clinicians to determine if further testing is warranted. APOE ε4 confers toxic functions or assesses the loss of neuroprotective functions that cause Alzheimer’s disease. According to Disease Control and Prevention (CDC) reports, the number of Alzheimer’s cases doubles every five years. According to World Health Organization reports, the number of cases will reach 152 million people with Alzheimer’s in 2050. The causes of AD are still unknown [5]. Still, some assumptions indicate that inside brain cells have accumulations of hyperphosphorylated proteins and outside brain cells have accumulations of peptide biomarkers called amyloid plaques [6]. Consequently, brain cell damage is caused by neurofibrillary tangles and amyloid plaques buildup [7]. It is essential to detect Alzheimer’s in its initial stage with the very mild or mild stages, called mild cognitive impairment (MCI), before the appearance of clinical biomarkers. The MCI stage is the stage of the onset of AD and is between health and injury [8]. People with this stage have slight changes in their cognitive abilities and can practice daily activities. About 20% of people over the age of 65% have MCI; in 35% of them, MCI progresses to AD within 3 to 5 years [9]. Thus, the stage of MCI either remains stable if detected early or will develop into AD. Structural and functional changes in AD have dynamic patterns [10] which occur early, before developing to AD. MRI imaging is a method to capture dynamic patterns, measure brain atrophy, and identify neurodegeneration [11]. Additionally, functional MRI measures changes in brain function, blood flow, and connectivity [12]. The MR spectroscopy is used to identify changes in brain receptors, such as gamma-aminobutyric acid (GABA) and glutathione (GSH) N-acetyl aspartate (NAA). MRI data cannot be obtained from images; therefore, the role of image reconstruction is to convert the acquired raw data into images that the doctor can interpret [13]. The process of converting the acquired data into images includes the steps of signal processing on modern MRI machines [14]. Brain imaging techniques provide a wide variety of information about the structure and tissues of the brain. Thus, MRI helped doctors evaluate functionally active brain areas for early diagnosis of AD [15]. Sometimes, one brain imaging technique does not contain enough information to diagnose AD. Furthermore, soft brain tissue is difficult to distinguish from healthy tissue by manual diagnosis. Manual extraction of the features of MRI images takes time, expertise, and effort. Therefore, diagnosis by artificial intelligence techniques is necessary to solve these challenges. MCI stages need accurate information to be identified. Therefore, deep learning models have sufficient capacity to extract the features of each stage of AD. This paper focused on developing systems capable of diagnosing AD in its first stage based on fused features, features extraction using deep learning models, and fusion features from more than one deep learning model, in addition to merging deep learning features with handcrafted features.

Because of the similarity of the biological and clinical characteristics of Alzheimer’s stages, it is difficult to distinguish between the stages of Alzheimer’s disease development. Features extraction and classification by CNN do not achieve satisfactory accuracy for distinguishing between Alzheimer’s stages. Furthermore, when extracting and classifying the handcrafted features, there is still a lack of satisfactory accuracy. Thus, this study contributed to distinguishing between Alzheimer’s stages and predicting each stage with high efficiency by combining CNN and handcrafted features.

The main contributions to this work are as follows:

- Improving MRI images of Alzheimer’s disease by overlapping between the average filter and the contrast limited adaptive histogram equalization (CLAHE) method.
- Combined the features of the GoogLeNet and Dense-121 models before and after the high-dimensionality reduction of the features and then fed them to the FFNN network to detect Alzheimer’s and predict its progression.
- Combined the features of the GoogLeNet and Dense-121 models separately with the handcrafted features and then fed them to FFNN to detect Alzheimer’s and predict its progression.
- Developing effective systems to help physicians and radiologists diagnose Alzheimer’s early and predict its progression.

The rest of this work has been organized as follows:

Section 2 discusses a range of previous studies for the early detection of Alzheimer’s disease. Section 3 presents methodologies and materials for analyzing MRI images of Alzheimer’s disease. Section 4 presents a summary of the performance results of the proposed systems to detect Alzheimer’s and predict its progression. Section 5 presents a discussion of the performance of all systems. Section 6 outlines the conclusions of this work.

## 2. Related Work

Here is a review of a group of previous research that includes techniques used to diagnose AD.

Modupe et al. presented the ResNet18 model to recognize AD in images of 138 subjects. The network performance was verified when setting the dropout of 0.2 and without the dropout; the network achieved better performance with a dropout of 0.2 for Alzheimer’s diagnosis. The system achieved a precision of 94%, recall of 90%, and an F1-score of 92% [16]. Samaneh et al. presented a framework capable of extracting biomarkers for the AD dataset to detect disease progression from mild cognitive to moderate cognitive and from moderate cognitive stage to Alzheimer’s. The framework achieved an average accuracy of 69% and precision of 73% [17]. Yingying et al. proposed a system to identify abnormal sequence changes in MR images to predict the transition of MCI to AD before clinical symptoms appear. A support vector machine (SVM) classifier was applied to detect the partial sequencing of MR images, which achieved an accuracy of 81.75% [18]. Selim et al. presented two homogeneous and heterogeneous methods for selecting the features of the AD dataset. The two groups of features were fed to many machine learning algorithms. The Random Forest (RF) algorithm produced the best performance among other algorithms for AD diagnosis with heterogeneous features with an accuracy of 91% [19]. Sambath et al. proposed a model with entropy image dissection and transfer learning. The entropy slicing method was applied to select the valuable slices during the training phase of the system to obtain segmented images. The pre-trained VGG-16 was used to train the AD dataset. The model had an accuracy of 93.12% [20]. Sharma et al. introduced a machine learning algorithm with feature extraction methods to support complementary features gained from neuroimaging techniques. Feature selection and merging methods were used to achieve high diagnostic efficiency [21]. Song et al. checked the performance of RF and other machine learning networks for AD dataset diagnostics. Feature selection methods were applied, and three feature groups were selected: 63, 29, and 22. The RF network achieved an accuracy of 90.2%, the multi-layer perceptron (MLP) network of 89.6%, and the convolutional neural network (CNN) of 90.5% with 63 features. On the other hand, when rating 22 features, the performance of the RF network increases, while the performance of the MLP and CNN networks decreases. They concluded that the RF network’s performance increases with fewer features [22]. Murugan et al. applied the CNN model to add a framework capable of analyzing MR images to discover the features of AD. The CNN generates high-resolution maps of the brain’s structure into a receptor that provides a visualization of the risk of AD. The model had an accuracy of 95.23% and an AUC of 97% [23]. Battineni et al. used six machine learning classifiers based on the features of MR images with patient characteristics. The gradient boosting algorithm achieved the best performance by 97.58% [24]. Badiea et al. proposed hybrid methods for Alzheimer’s diagnosis. The classification layer of CNN models has been replaced by the SVM algorithm. The system had an accuracy of 94.8% and a sensitivity of 97.75% [25]. Sun et al. developed ResNet based on Spatial Transformer Networks (STN) for early diagnosis of AD. The Mish activation function was set for the ResNet-50 model, and the STN was inserted into the ResNet-50 layers. The spatial information of the MR images was converted into basic information spaces through STN. The developed network can solve the information loss problem in CNN; the network achieved an accuracy of 95.5% [26]. Duaa et al. introduced a pre-trained ResNet50 network to extract the features of MR images for diagnosing AD. The performance of ResNet50 was evaluated using the Softmax activation function and the SVM and RF algorithms. ResNet50 with SVM achieved 92% accuracy and 91% specificity [27].

All previous studies aimed to achieve high accuracy in distinguishing between the stages of progression of AD. Because of the similar features of MR images in the early stages and the low contrast between soft and healthy brain cells, this study focused on extracting the hybrid features by several methods. In this study, features are extracted by two CNN and combined with some feature vectors. The CNN model features are combined with the handcrafted features extracted by many methods, namely DWT, LBP and GLCM.

## 3. Methods and Materials

**Figure 1:**
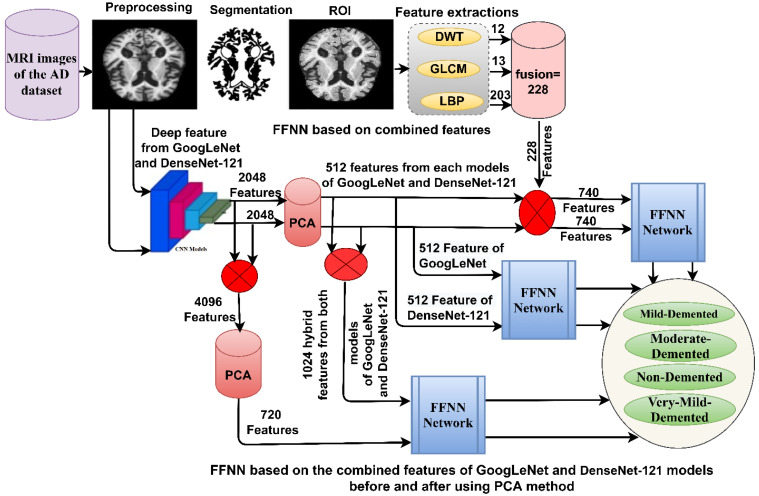
Methodology framework for diagnosing MRI images for early detection of AD progression stages.

The section provides materials for the diagnosis of MRI images of Alzheimer’s disease for early detection of its stages of development, as shown in Figure 1. MRI images of Alzheimer’s disease were improved with the same method for all systems and then fed to the GoogLeNet and Dense-121 models. The first methodology is by FFNN with the features of the GoogLeNet and Dense-121 models. The second method is by FFNN with the combined features between the GoogLeNet and Dense-121 models before and after the high dimensionality of the features was reduced by PCA. The third methodology is by FFNN with features combined between GoogLeNet and Dense-121 models separately with handcrafted features.

### 3.1. Description of the MRI Dataset

**Figure 2:**
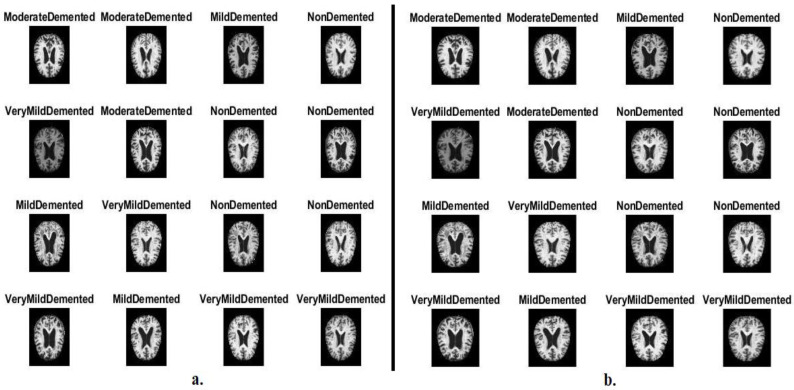
MRI image samples for the AI dataset (**a**) before improved images and (**b**) after improved image.

In this study, systems have been developed that have superior abilities for early detection of AD by evaluating MRI images of the AD dataset. The data were obtained from different websites verifying both classes and labels. The AD dataset consists of 6400 images distributed between four unbalanced classes: three types of Alzheimer’s progression and a normal class of MRI. Each class in the Alzheimer’s dataset consists of the following images: mild_dementia of 896 MRI, moderate_dementia of 64 MRI, non_dementia of 3200 MRI, and very_mild_dementia of 2240 MRI. Figure 2a shows samples from the Alzheimer’s dataset [28].

### 3.2. Enhancement of MRI Images

MRI images are affected by some noise due to different reasons, such as patient movement when undergoing MRI images, brightness, reflections, low contrast, and obtaining MRI images from several devices; all of these reasons affect the performance of the proposed systems. Thus, improving MRI images leads to enhanced images, which helps obtain high performance [29]. This study applied two techniques, which are average filter and CLAHE, to improve MRI images.

First, we enhanced the MRI images using the average filter. According to the filter method, the filter works every time by changing a pixel with an average of 15 adjacent pixels. The average of 15 adjacent pixels is calculated and replaced in place of the central pixel [30]. Equation (1) shows the candidate’s work, which continues to work until the completion of the image:

(1) $$F(z)=\frac{1}{N}\sum_{i=0}^{N-1}y(z-1)$$

where *F*(*z*) is the improved image, *y*(*z* − 1) is the past input, and *N* is the pixels on the image.

Second, low-region contrast has been increased for MRI images by the CLAHE technique. The method evenly distributes the light pixels of the MRI images, which improves the contrast of the edges of the affected areas. The method takes a pixel each time and changes it with another pixel based on the transformation function. The pixels adjacent to each central pixel are taken according to the image’s contrast. When the goal pixel value is smaller than the neighbor’s pixels, the contrast of the image will increase, while when the goal pixel value is greater than the neighbor’s pixels, the contrast of the image will decrease [31]. Thus, MRI images were improved. Figure 2b shows an optimized MRI image sample from the Alzheimer’s dataset from all classes.

Finally, the image improved for the average filter was combined with the image improved for the CLAHE method to obtain a more improved image.

### 3.3. Evaluating Systems

The performance results for all systems on the AD dataset were obtained using the same evaluation scales. Equations (2) through (8) show the systems evaluation scales; the equations contain variables, such as TN and TP, which means adequately categorized images, and FN and FP, which means improperly categorized images. All equation variables are extracted from the confusion matrix [32]:

(2) $$Accuracy=\frac{TN+TP}{TN+TP+FN+FP}*100\%$$

(3) $$Sensitivity=\frac{TP}{TP+FN}*100\%$$

(4) $$Precision=\frac{TP}{TP+FP}*100\%$$

(5) $$Specificity=\frac{TN}{TN+FP}*100$$

Given a threshold *s*, the instance is classified as “positive” if *X* > *T*, and “negative” otherwise:

(6) $$TPR\left(T\right)=\int_{T}^{\infty }f_{1}\left(x\right)dx$$

(7) $$FPR\left(T\right)=\int_{T}^{\infty }f_{0}\left(x\right)dx$$

ROC curve plots parametrically *TPR*(*T*) versus *FPR*(*T*) with *T* as the varying parameter.

When using standardized units, the AUC is equal to the probability that a classifier will classify a randomly chosen positive image higher than a negative one:

(8) $$AUC(F)=\frac{\sum_{t_{0}\in {(D}^{0}}\sum_{t_{1}\in {(D}^{1}}1\left[f\left(t_{0}\right)<f\left(t_{1}\right)\right]}{\left|D^{0}\right|.\left|D^{1}\right|}$$

where $1\left[f\left(t_{0}\right)<f\left(t_{1}\right)\right]$ represents function which returns 1 if $f\left(t_{0}\right)<f\left(t_{1}\right)$ otherwise return 0, $D^{0}$ is the set of negative images, and $D^{1}$ is the set of positive images.

### 3.4. Data Augmentation and Balancing Dataset

**Figure 3:**
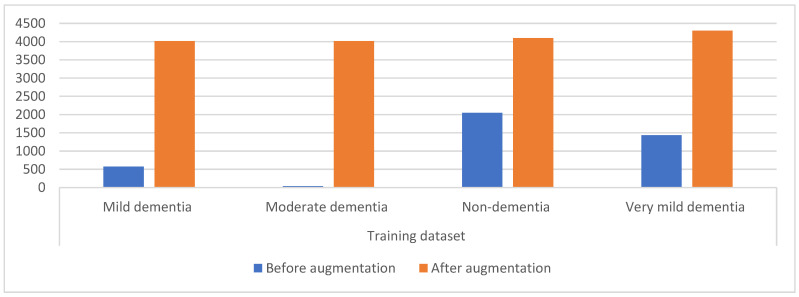
Displaying the distribution of MRI images for the AD dataset before and after applying the data augmentation.

A limitation of CNN models is a dataset that contains few images. CNN needs a dataset with many images in order to be able to train the systems and fill them with sufficient information during the training phase. Additionally, the unbalanced dataset affects the performance of CNN and is considered one of the limitations for the reason the accuracy is inclined to the class that contains images more (the majority). Therefore, the data augmentation technique solves the mentioned challenges by increasing the images artificially of the dataset from the same images of the dataset. To solve the unbalanced dataset, the data augmentation technique produces images from the class of minority by a more significant percentage than the increase in the majority class. Thus, this technique increases the images by balancing the dataset. Many operations augment the dataset images, such as shifting, flipping, rotating with many angles, and others [33]. Figure 3 shows the distribution of MRI images among the dataset classes, where the image numbers before applying the data increase are very few and unbalanced. The figure also shows the distribution of MRI images between the dataset classes after applying the data augmentation. Table 1 describes the distribution of MRI images between the dataset classes during training before and after using the data augmentation. It is noted from the table that the mild dementia class was increased by six artificial images from each image, for the moderate dementia class, each image was increased by 97 images artificially, and the non-dementia class was increased by one image artificially from each image; finally, for the very mild dementia class it was increased by two images artificially from each image.

### 3.5. FFNN Network According to the Features of CNN Models

When training a dataset using CNN models, there are some challenges, such as requiring high-spec computers, being expensive, time-consuming, and not achieving satisfactory results for diagnosing some datasets. Thus, the hybrid technique between CNN and the FFNN solves this challenge. The technique contains two parts: the first is CNN for extracting features from MRI images of AD, and the second is the FFNN algorithm to diagnose the extracted features. The PCA method is used after CNN to reduce the high dimensionality of features.

#### 3.5.1. Extracting the Deep Features

CNN models are distinguished from machine learning networks in that they consist of many layers to extract deep feature maps without manual intervention. In this work, the GoogLeNet [34] and DenseNet-121 [35] models were fed with MRI images improved for extracting features through several convolutional layers. Each convolutional layer contains thousands of neurons connected by weights, connections, and complex mathematical operations. MRI images pass through convolutional, auxiliary, and pooling layers to extract deep features.

Convolutional layers are one of the CNN layers, the number of which varies from one network to another. The task of convolutional layers is to extract features, and their tasks differ from one layer to another [36]. Each layer performs a specific action, for example, a layer that extracts shape features, another that extracts color features, a layer that shows edges, and so on. There are three main parameters that control the work of each layer; first, the filter size *f*(*t*), which determines the pixels that wrap around the target image *x*(*t*) as in Equation (9). Second, the p-step, which determines the number of steps the filter slides over the image. Third, the zero-padding of the image edges, which preserves the original image size during the processing process [37]:

(9) $$W\left(t\right)=\left(x*f\right)\left(t\right)=\int x\left(a\right)f\left(t-a\right)da$$

where *W*(*t*) is the layer of output, *f*(*t*) is the filter and *x*(*t*) is the MRI image inputted.

CNN models provide pooling layers that reduce the high dimensionality of features produced by convolutional layers, causing time to train and require complex computations. Pooling layers work in two methods, the max–average pooling methods [38]. The max pooling method selects a set of pixels, chooses the max pixel, and replaces it with the selected pixels as in Equation (10). The average pooling method selects a set of pixels, calculates its average, and replaces it with the selected pixels as in Equation (11):

(10) $$z\left(i;\;j\right)={max}_{m,n=1\ldots .k}\;f\left[\left(i-1\right)p+m;\;\left(j-1\right)p+n\right]$$

(11) $$z\left(i;\;j\right)=\frac{1}{k^{2}}\sum_{m,n=1\ldots .k}\;f\left[\left(i-1\right)p+m;\;\left(j-1\right)p+n\right]$$

where *m*, *n* means the position in the matrix, *f* is the filter size, *p* is the step filter, and *k* is the vectors of the features.

The features are extracted for the GoogLeNet and Dense-121 models and saved in a features matrix of size 6400 × 2048 for each model. The GoogLeNet and Dense-121 models follow the PCA method to reduce the high dimensionality of the features and save them at a size of 6400 × 512 for each model and then feed them to the FFNN network.

#### 3.5.2. FFNN Network

**Figure 4:**
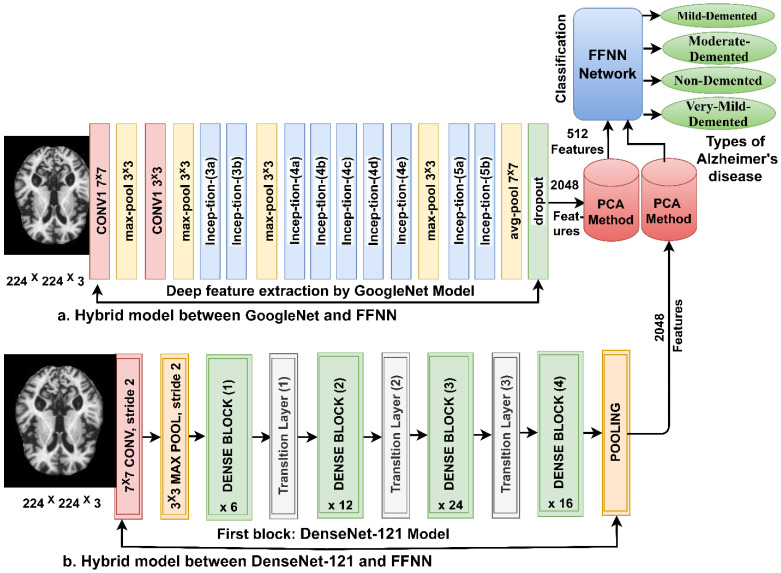
Basic methodology for diagnosing MRI images of the AD by FFNN according to CNN features.

FFNN is a high-performance neural network to solve many tasks, such as the classification of images. FFNN consists of three layers; first, the input layer has several units as the size of the features vector. Second, the hidden layers include many layers connected to specific weights. Complex operations are performed in hidden layers to perform particular tasks. The output layer contains as many neurons as the number of the dataset classes [39]. The FFNN feeds the features to the forward, where each new neuron produces the output of the previous neuron with the weight of them. FFNN updates the weights on each iteration and calculates a minimum quadratic error (MSE between the expected values *y* and the actual values *x* as in Equation (12). In this work, FFNN has 512 units in the input layer, 10 hidden layers, and 4 neurons in the output layer. Figure 4 shows the framework of the technique for diagnosing MRI images of AD. It is noted that the method contains two models of CNN, dimension reduction using PCA and low-dimension features diagnosis by FFNN [40]:

(12) $${MSE=\frac{1}{N}\sum_{j=1}^{N}\left(x_{j}-y_{j}\right)}^{2}$$

where *N* means the number of data points, $x_{j}$ means the actual output, and $y_{j}$ means the predicted output.

### 3.6. FFNN Network According to Fusing Features of CNN Models

**Figure 5:**
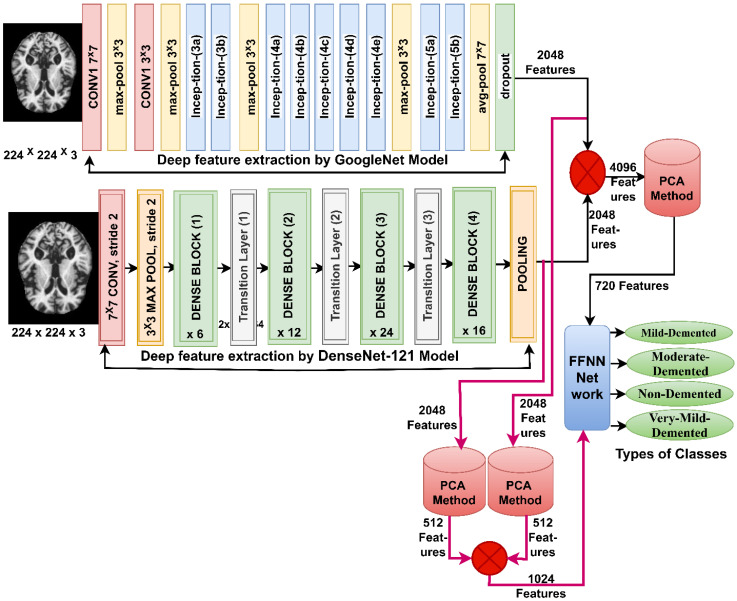
Basic methodology for diagnosing MRI images of the AD dataset by FFNN according to the fusion of CNN features.

When one of the CNN models is used to diagnose a dataset, it does not achieve satisfactory results with some datasets and is time-consuming with an expensive computer. Thus, this section presents a new technique for diagnosing MRI images by FFNN network [41] based on integrating the features of the GoogLeNet and Dense-121 models and then reducing the high dimensions and merging them into the same features vector, and reducing the high dimensions of the features of the GoogLeNet and Dense-121 models and then combining them into the same features vector [42]. This technique consists of two systems shown in Figure 5.

The first system consists of the following steps: first, the MRI images of the AD dataset are improved and passed to the GoogLeNet and Dense-121 models. Second, the convolutional layers of the GoogLeNet and Dense-121 models extract the features of the MRI images and store them in a features matrix of size 6400 × 2048 for each model. Third, the features of both GoogLeNet and Dense-121 models are combined in the same vector and saved in a features matrix of size 6400 × 4096. The fourth step involves passing the features matrix to the PCA method to reduce the high dimensionality of the features [43], PCA produces a features matrix of size 6400 × 720. The fifth step passes the features matrix of size 6400 × 720 to the FFNN network to train them and evaluate FFNN performance at high speed and effective efficiency.

The steps for implementing the second system are as follows: the first and second steps are the same as the steps of the first system. Third, passing the features of a GoogLeNet model size of 6400 × 2048 to the PCA method reduces the high dimensions and saves them in a feature array of size 6400 × 512. The fourth step passes the features of the DenseNet-121 model size of 6400 × 2048 to PCA to reduce the high dimensions and save them in a features matrix of 6400 × 512. The fifth step combines the features of the GoogLeNet and Dense-121 models after reducing the high dimensions and saving them into a features matrix of size 6400 × 1024. The sixth step passes the features matrix of size 6400 × 1024 to the FFNN network to train them and evaluate the performance of the FFNN at high speed and effective efficiency.

These systems aim to diagnose images of MRI of AD using FFNN according to a mixture of features between GoogLeNet and Dense-121 models. The integration of features is before and after the application of PCA technique.

### 3.7. FFNN Network According to Fusing the Features of CNN Models with Handcrafted Features

**Figure 6:**
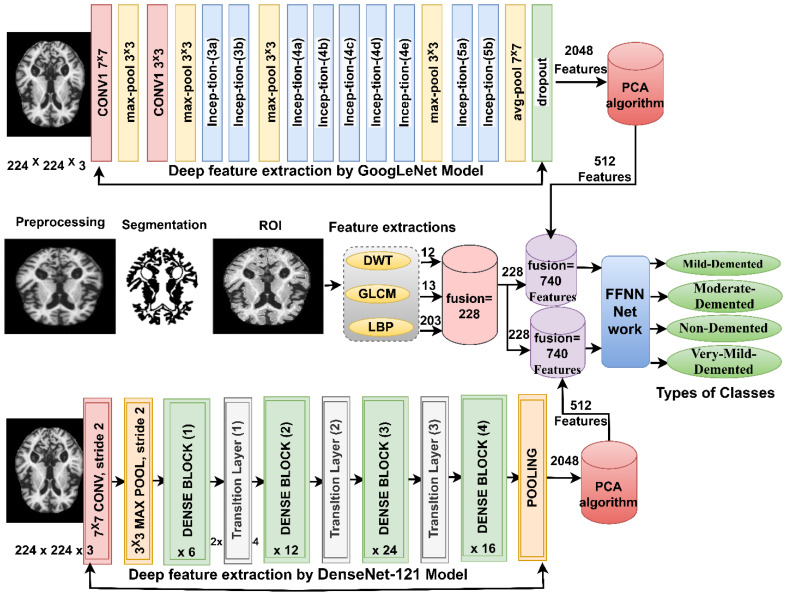
Basic methodology for diagnosing MRI images of the AD dataset by FFNN according to the fusion of CNN features and handcrafted feature.

The section presents a modern methodology for MRI image diagnosis of AD dataset by FFNN based on extracting features from GoogLeNet and Dense-121 models separately and then combining them with features of DWT, LBP and GLCM methods (handcrafted features) [44]. This technique consists of two systems shown in Figure 6.

The first system consists of the following steps: first, the images of MRI of the AD dataset are optimized and passed to the GoogLeNet and Dense-121 models. Second, the convolutional layers of the GoogLeNet and Dense-121 models extract the features of the MRI images and store them in a features matrix of size 6400 × 2048 for each model separately. Third, passing the two feature matrices to the PCA method to reduce the high dimensionality of the features, the PCA method produces two feature matrices of size 6400 × 512 for each model separately. Fourth, the optimized MRI images are passed to the DWT, LBP and GLCM algorithms, and each algorithm produces features.

The DWT method analyzes the image into four parts (components); three features are extracted from each part using statistical measures. The algorithm analyzes each part of the image through a specific filter [45]. A low-pass filter analyzes the first part of the image; the filter analyzes the approximate parameters of the first part of the image and extracts three features: standard deviation, variance, and mean. The second and third parts of the image are analyzed by Low–High and High–Low filters; the candidates analyze the detailed parameters from the second and third parts of the image and extract six features, the standard deviation, variance, and the mean, from each part of the image. The high pass filter analyzes the fourth part of the image; the filter analyzes the detailed parameters of the fourth part of the image and extracts three features: standard deviation, variance, and mean. Thus, the DWT method produces 12 features stored in a features matrix of size 6400 × 12.

The GLCM method converts an image’s region of interest into a gray matrix to extract texture features. The algorithm collects spatial information from a region of interest and extracts features, so that GLCM compares each pixel based on the distance d to its neighbor and the angles 0°, 45°, 90° and 135° between the pixel and the neighbor. After analyzing the region of interest in the image, the algorithm detects whether it is a smooth or rough region [46], so that the rough region contains unequal pixels, while the smooth region contains equal pixels. The features extracted by GLCM are Correlation, Contrast, Homogeneity, Energy, Entropy, Mean, Standard-Deviation, Variance, RMS, Skewness, Kurtosis, Dissimilarity, and Smoothness. Thus, the GLCM method produces 13 features stored in a features matrix of size 6400 × 13.

The LBP method converts the image into a gray matrix to extract the features of the binary surface texture. The algorithm describes the MRI texture of AD by measuring local contrast. The size of the method is set to 5 ∗ 5. Thus, the method selects 16 pixels; 1 pixel at a time is chosen as the central pixel $g_{c}$, and 24 pixels as the neighbor pixels $g_{P}$. The central pixel is replaced by the output of the LBP method according to the analysis of the adjacent 24 pixels, as illustrated by Equation (13). The mechanism continues until each pixel in the image is targeted and replaced with its neighbors according to the algorithm’s working mechanism [47]. Thus, the LBP method produces 203 features saved in a features matrix of size 6400 × 203:

(13) $${LBP}_{R,P}=\sum_{n=0}^{P-1}s\left(g_{P}-g_{c}\right)2^{n}$$

where $g_{c}$ is the target pixel, $g_{P}$ is neighbor pixels *R* is the neighbor radius, and *P* is the number of neighbor pixels.

Fifth, the features of the three algorithms are combined into a features matrix of size 6400 × 228.

Sixth, the two feature matrices which result after dimensionality reduction by PCA method are combined with the handcrafted features. Thus, the features matrix size is 6400 × 740 for the GoogLeNet and handcrafted feature and the features matrix size is 6400 × 740 for the DenseNet-121and handcrafted feature. The seventh step involves passing the two feature matrices of size 6400 × 740 to the FFNN network to train them and evaluate the performance of the FFNN at high speed and effective efficiency.

## 4. Results of the Performance of the Systems

### 4.1. Results of FFNN Network According to the Features of CNN Models

This section presents FFNN results according to the features extracted from the GoogLeNet and Dense-121 models of MRI image analysis for early AD detection. This technique is based on extracting the features from the GoogLeNet and Dense-121 models separately, then reducing the dimensions of the high features by the PCA method and saving them in two feature matrices. The two feature matrices have been fed separately to the FFNN, which divides the features matrix into 80% for training and validation of the FFNN and 20% for testing the performance of the FFNN.

Table 2 presents the FFNN results according to the features of the GoogLeNet and Dense-121 models separately. FFNN with the features of the GoogLeNet model performed slightly better than FFNN with the features of the DenseNet-121 model. The performance of FFNN with GoogLeNet features reached an accuracy of 94.8%, sensitivity of 87.66%, AUC of 92.54%, precision of 92.08%, and specificity of 98.25%. In contrast, the performance of FFNN with DenseNet-121 features reached an accuracy of 93.6%, sensitivity of 85.91%, AUC of 93.31%, precision of 89.35%, and specificity of 97.63%.

**Figure 7:**
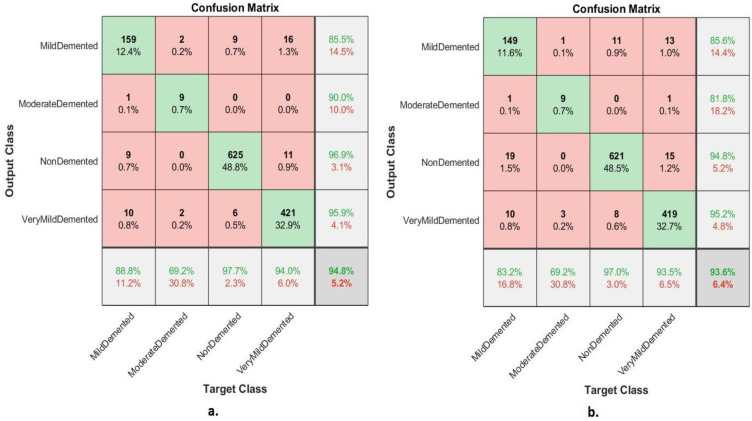
Confusion matrix of FFNN performance for detection of AD based on features (**a**) GoogLeNet and (**b**) DenseNet-121.

The confusion matrix shown in Figure 7 shows the performance of FFNN according to the features of the GoogLeNet and Dense-121 models for the detection of AD stages. The FFNN with GoogLeNet features achieved accuracy for each class as follows: mild demented of 88.8%, moderate demented of 69.2%, non-demented of 97.7% and very mild demented of 94%. In contrast, the FFNN with DenseNet-121 features achieved accuracy for each class as follows: mild demented of 83.2%, moderate demented of 69.2%, non-demented 97% and very mild demented of 93.5%.

### 4.2. Result of FFNN According to Fusing the Features of CNN Models

This section presents FFNN results based on GoogLeNet features combined with DenseNet-121 features for analyzing MRI detection of AD progression. This technology consists of two approaches based on integrating the features of GoogLeNet and DenseNet-121. The first approach is based on combining the features of GoogLeNet and DenseNet-121 and saving them in a features matrix, then feeding them into the PCA to reduce the high dimensionality of the features. Then, feeding the features matrix to FFNN, which divides the features matrix into 80% for training and validation of FFNN and 20% for testing the FFNN. The second approach is based on extracting GoogLeNet features, reducing high dimensionality by PCA, and saving them in a low-dimensional features matrix and, at the same time, extracting DenseNet-121 features and reducing high dimensionality by PCA and saving them in a low-dimensional features matrix. Then, merging the two low-dimensional feature matrices into one feature matrix. Then, feeding the features matrix to FFNN, which divides the features matrix into 80% for training and testing the FFNN.

Table 3 present the FFNN results according to the features of the merged GoogLeNet and Dense-121 models before high-dimensionality reduction of the features and by merging after high dimensional reduction of the features of the two models. The performance of FFNN with the combined features of the GoogLeNet and Dense-121 models after high-dimensionality reduction was slightly better than the performance of FFNN with the combined features of the GoogLeNet and Dense-121 models before the high-dimensionality reduction. The performance of FFNN with the combined features of GoogLeNet and Dense-121 models before high-dimensionality reduction had an accuracy of 97.2%, sensitivity of 89.95%, AUC of 94.25%, precision of 89.69%, and specificity of 99.1%. In contrast, the performance of FFNN with the combined features of GoogLeNet and DenseNet-121 model after high-dimensionality reduction had an accuracy of 98.8%, sensitivity of 96.5%, AUC of 97.06%, precision of 96.68%, and specificity of 99.54%.

**Figure 8:**
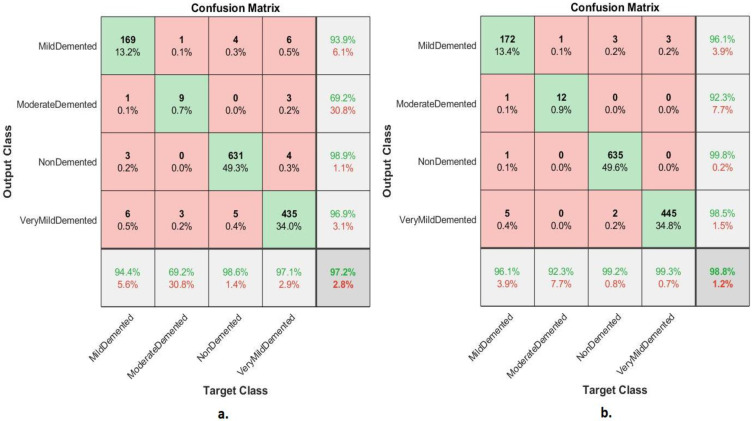
Confusion matrix of FFNN performance for AD detection based on combined features of the GoogLeNet and DenseNet-121 (**a**) before PCA and (**b**) after PCA.

The confusion matrix shown in Figure 8 illustrates the performance of the FFNN according to the feature fusion of the GoogLeNet and Dense-121 models before and after using the PCA method for detection of Alzheimer’s progression. The FFNN with combined features of the GoogLeNet and Dense-121 models before reducing the high dimensionality achieved accuracy for each class of 94.4%, 69.2%, 98.6%, and 97.1% for mild, moderately dementia, non-dementia, and very mild, respectively. In contrast, the FFNN with feature combined of GoogLeNet and Dense-121 models after high-dimensionality reduction achieved accuracy for each class of 96.1%, 92.3%, 99.2 and 99.3% for mild, moderate, non-demented, and very mild dementia, respectively.

### 4.3. Results of FFNN According to Fusing the CNN Features with Handcrafted Features

This section presents the performance of FFNN based on the combined features of CNN models and the handcrafted features of MRI image analysis for AD detection progression. This system consists of two approaches that rely on features combined between CNN models and handcrafted features. The first approach combines GoogLeNet features with handcrafted features and stores them in a features matrix. This is followed by feeding the features matrix to FFNN, which divides the features matrix into 80% for training and validation of FFNN and 20% for testing the FFNN. The second approach combines DenseNet-121 features with handcrafted features and stores them in a features matrix. This is followed by feeding the features matrix to FFNN, which divides the features matrix into 80% for training and validation of FFNN and 20% for testing the FFNN. The technology provides some tools for evaluating FFNN on the Alzheimer’s dataset.

#### 4.3.1. Confusion Matrix

The section presents a confusion matrix to evaluate FFNN performance by combining CNN features with handcrafted features for diagnosis of AD progression. Because of the similarity between MRI images during the AD progression, the features play a crucial role in distinguishing between the stages of AD. Thus, in this section, the deep features of the GoogLeNet model are combined with the handcrafted features and saved in a features matrix, and the deep features of the DenseNet-121 model are combined with the handcrafted features and saved in a features matrix. Finally, FFNN was fed with two features matrixes, which divided features matrix into 80% for training and validation of the FFNN and 20% for performance of the FFNN.

**Figure 9:**
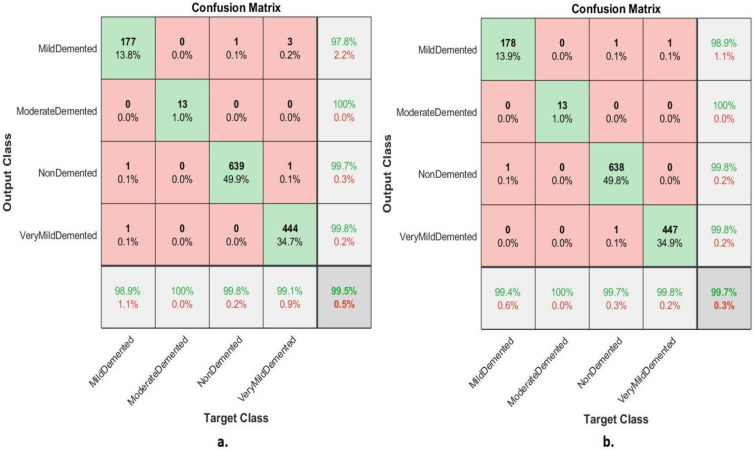
Confusion matrix of FFNN performance for MRI for detection of AD based on combined features between (**a**) GoogLeNet and handcrafted features and (**b**) DenseNet-121 and handcrafted features.

Figure 9 shows the confusion matrix of FFNN’s performance for the AD detection progression. The FFNN by combined features between GoogLeNet and handcrafted achieved accuracy for each class of 98.9%, 100%, 99.8%, and 99.1% for mild, moderately dementia, non-dementia, and very mild, respectively. In contrast, the FFNN by combined features between GoogLeNet and handcrafted achieved accuracy for each class of 99.4%, 100%, 99.7%, and 99.8% for mild, moderately dementia, non-dementia, and very mild, respectively.

Table 4 presents the FFNN results according to the combined features between the CNN models and the handcrafted features. The FFNN with combined features between the DenseNet-121 model and handcrafted features performed slightly better than the FFNN with combined features of the GoogLeNet model and handcrafted features. FFNN with combined features between the GoogLeNet model and handcrafted features achieved an accuracy of 99.5%, sensitivity of 99.39%, AUC of 99.17%, precision of 99.33%, and specificity of 99.7%. In contrast, the performance of FFNN with the hybrid features of the DenseNet-121 and the handcrafted features had an accuracy of 99.7%, sensitivity of 99.64%, AUC of 99.56%, precision of 99.63%, and a specificity of 99.67%.

#### 4.3.2. Error Histogram

**Figure 10:**
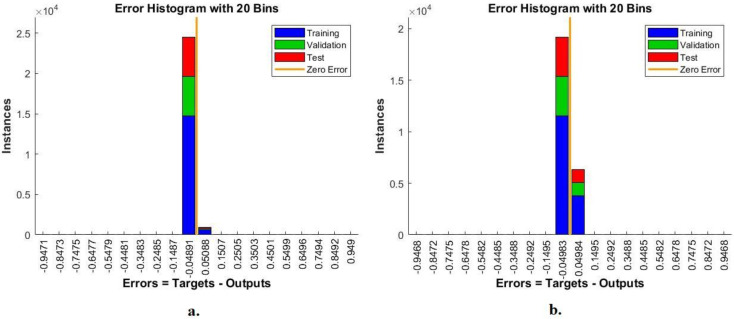
Display error histogram for diagnosing MRI for detection of Alzheimer’s progression by FFNN with combined features of (**a**) GoogLeNet and handcrafted features and (**b**) DenseNet-121 and handcrafted features.

The FFNN network was evaluated on the Alzheimer’s dataset through the error histogram tool to detect the early stages of Alzheimer’s progression. The error histogram checks the error between the output and target values. It registers errors according to instances during the dataset’s training, validation, and performance testing phase. The error histogram tool shows the network performance with a special color during each phase. Figure 10 shows an error histogram for evaluation of the FFNN during the diagnosis of MRI of AD. The blue color shows the performance of the FFNN during the training phase, the green color during the FFNN validation and adjustment of weights, and the red color during the testing of new MRI to evaluate the FFNN network. FFNN, with combined features between GoogLeNet and handcrafted features, achieved the best performance among 20 bins with −0.9471 and 0.949 values. In contrast, FFNN with combined features between DenseNet-121 and handcrafted features achieved the best performance among 20 bins with −0.9468 and 0.9468 values.

#### 4.3.3. Cross-Entropy

**Figure 11:**
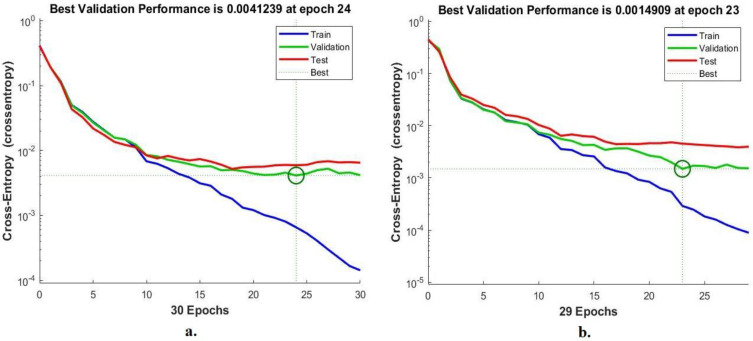
Display cross-entropy for diagnosing MRI for detection of Alzheimer’s progression by FFNN with combined features of (**a**) GoogLeNet and handcrafted features and (**b**) DenseNet-121 and handcrafted features.

FFNN was evaluated on the Alzheimer’s dataset by a cross-entropy tool to detect early stages of Alzheimer’s progression. Cross-entropy checks the error between actual and expected values. It registers the error in each epoch during all phases and the FFNN performance testing phase. The cross-entropy tool shows the network performance during each phase with a special color. Figure 11 shows cross-entropy for the evaluation of the FFNN during the diagnosis of the MRI dataset. The blue color shows the performance of the FFNN during the training phase, the green color during the FFNN validation and adjustment of weights, and the red color during the selection of new MRI images to evaluate the FFNN network. With the combined features of GoogLeNet and the handcrafted features, the FFNN obtained the best performance through the intersection of the dashed lines represented by a small circle at the epoch of 24, where the network reached a minimum error of 0.0041239. In contrast, when combining features of DenseNet-121 and handcrafted features, the FFNN achieved the best performance at the epoch of 23, when the network reached a minimum error of 0.0014909.

#### 4.3.4. Gradient and Validation Checks

**Figure 12:**
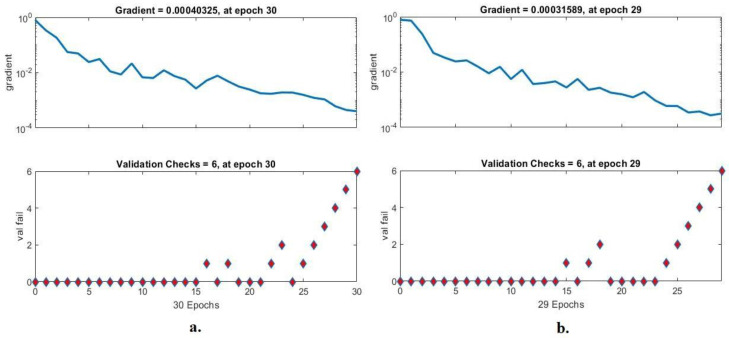
Display gradient and validation checks for recognizing MRI of Alzheimer’s progression by FFNN with combined features of (**a**) GoogLeNet and handcrafted features and (**b**) DenseNet-121 and handcrafted features.

FFNN was evaluated on the Alzheimer’s dataset through gradient and validation checks to detect early stages of Alzheimer’s progression. Gradient checks network performance during each epoch, and the validation checks for checking failed values during each epoch. Figure 12 shows the gradient for the evaluation of FFNN during the diagnosis of the MRI dataset. With combined features between GoogLeNet and handcrafted features, the FFNN achieved the best performance at epoch 30 with a gradient of 0.00040325 and a validation value of 6. In contrast, when combining features between DenseNet-121 and handcrafted features, the FFNN achieved the best performance at epoch 29 with a gradient of 0.00031589 and validated with a value of 6.

### 4.4. Results of Systems Generalization on the ADNI Dataset

This section presents a generalization of FFNN performance based on the combined features of CNN models and handcrafted MRI image analysis of a new dataset called Alzheimer’s Disease Neuroimaging Initiative (ADNI) for AD detection. The ADNI dataset consists of five classes: Alzheimer’s disease (AD), cognitively normal (CN), mild cognitive impairment (MCI), early MCI (EMCI), and late MCI (LMCI).

Table 5 presents the FFNN results based on hybrid features of GoogLeNet-Handcrafted and DenseNet121-Handcrafted extracted from the ADNI dataset. FFNN with merged features between the GoogLeNet and handcrafted features achieved an accuracy of 92.3%, sensitivity of 87.22%, AUC of 91.2%, precision of 89.82%, and specificity of 97.92%. In contrast, the performance of FFNN with the merged features of the DenseNet-121 and the handcrafted features has an accuracy of 93.4%, sensitivity of 90.88%, AUC of 94.34%, precision of 88.98%, and a specificity of 98.42%.

**Figure 13:**
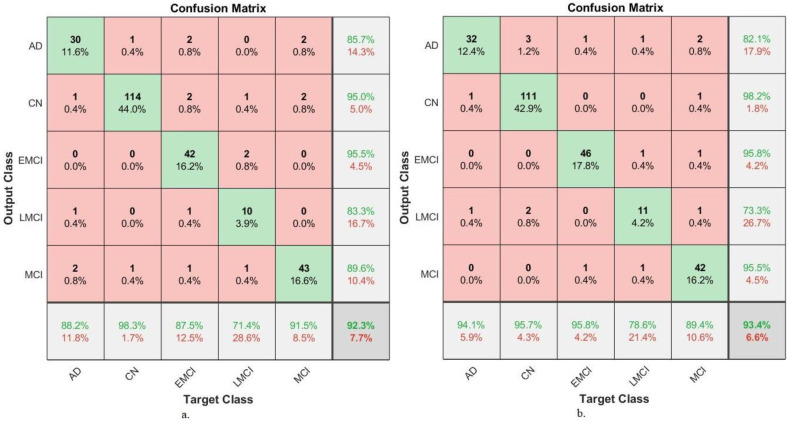
Confusion matrix for generalization performance of FFNN for MRI image analysis of an ADNI dataset based on fused features between (**a**) GoogLeNet and handcrafted and (**b**) DenseNet-121 and handcrafted.

Figure 13 displays the confusion matrix of FFNN performance for the detection of AD. The FFNN, by combining features of GoogLeNet and handcrafted, reached accuracy for each class: AD class of 88.2%, CN class of 98.3%, EMCI class of 87.5%, LMCI class of 71.4% and MCI class of 91.5%. In contrast, the FFNN, by combining features of GoogLeNet and handcrafted reached accuracy for each class: AD class of 94.1%, CN class of 95.7%, EMCI class of 95.8%, LMCI class of 78.6% and MCI class of 89.4%.

## 5. Discussion of the Performance of Approaches

This work presented the development of many diverse systems with different materials to reach superior results for the early detection of each stage of AD. Because of the similarity of MRI images in the early stages of Alzheimer’s and the similarity between healthy and soft brain cells, the systems focused on extracting the features from several methods and integrating them. Initially, MRI images were optimized using the same technique for all systems. This paper discusses three methodologies, each with two systems for detection of AD progression. The first methodology for detection of AD progression stages by FFNN based on the features of GoogLeNet and DenseNet-121 separately. FFNN with GoogLeNet features achieved 94.8% accuracy; in contrast, FFNN with DenseNet-121 features achieved an accuracy of 93.6%.

The second methodology was for detection of AD progression by FFNN with integrated features of GoogLeNet and Dense-121 models before and after high-dimensionality reduction. With the combined features of GoogLeNet and DenseNet-121 and then reducing the high dimensionality of the features, FFNN achieved an accuracy of 97.2%. In contrast, FFNN achieved an accuracy of 98.8% with the combined features between GoogLeNet and DenseNet-121 after reducing the high dimensionality of the features.

The third methodology for detection of AD progression stages by FFNN based on features combined between GoogLeNet and handcrafted features, in addition to the same network with features combined between the DenseNet-121 and handcrafted features. With the combined features of GoogLeNet and handcrafted features, FFNN achieved an accuracy of 99.5%; in contrast, FFNN achieved an accuracy of 99.7% with the combined features of GoogLeNet and handcrafted features.

Table 6 summarizes all systems to diagnose images of MRI for classifying AD progression. The table shows the accuracy for each system in addition to the accuracy for each class of the dataset for all systems. The best accuracy of 99.7% by FFNN with combined features of DenseNet-121 and handcrafted features. For mild dementia and very mild dementia classes, the accuracy of their diagnosis was 99.4% and 99.8% using FFNN with combined features of DenseNet-121 and handcrafted features. For the moderate dementia class, the accuracy of diagnosis was 100% using both the FFNN network with the combined features of DenseNet-121 and handcrafted features, an addition to the FFNN with the combined features of DenseNet-121 and handcrafted features. For the non-dementia class, it was diagnosed with an accuracy of 99.8% using FFNN with the combined features of DenseNet-121 and handcrafted features.

The proposed systems have the superior ability to distinguish between two different stages, whether for other patients or the same patient. If the patient was diagnosed with a specific stage, and after a period, Alzheimer’s developed for the same patient, and they underwent MRI and was fed to the automated system for evaluation, then the system has the ability to select any stage of Alzheimer’s [48].

The methods and materials applied in this study were varied, which focused mainly on the gap found in previous studies represented in combining features to solve the problem of similarity of characteristics in Alzheimer’s stages. Thus, through the results of previous studies, it is noted that the results achieved by the proposed systems surpassed the results of previous relevant studies.

## 6. Conclusions

AD is one of the diseases for which there is no cure, and early prediction is an effective method to slow its progression to the late stage, which causes brain cell damage and death. This paper contributed to developing many methodologies to detect Alzheimer’s disease and predict its progression. This study focused on integrating the features of several methods into a matrix of features and feeding them to the FFNN network to diagnose them. The first methodology uses the FFNN with the features of the GoogLeNet and Dense-121 models. The second methodology uses the FFNN network with features combined between GoogLeNet and Dense-121 models before and after reducing the high dimensionality of the features by using PCA. The third methodology uses the FFNN with features combined between GoogLeNet and DenseNet-121 model separately with handcrafted features extracted by DWT, LBP and GLCM methods. FFNN achieved an accuracy of 99.7%, sensitivity of 99.64%, AUC of 99.56%, precision of 99.63%, and a specificity of 99.67%. with hybrid features of the DenseNet-121 model and the handcrafted features.

The limitations that we faced were in the scarcity of sufficient images to train the dataset, which was processed by the method of data augmentation. Furthermore, the imbalance of the classes is one of the limitations that we faced, as the accuracy tended to the majority class, which was treated by the data augmentation method.

## Figures and Tables

**Table 1:** The technique of balancing and augmentation of MRI image data for AD dataset.

Phase	Training Dataset			
Classes	Mild Dementia	Moderate Dementia	Non-Dementia	Very Mild Dementia
Before augmentation	574	41	2048	1434
After augmentation	**4018**	**4018**	**4096**	**4302**

**Table 2:** FFNN results with features of GoogLeNet and DenseNet-121.

Models	Classes of AD	Accuracy %	Sensitivity %	AUC %	Precision %	Specificity %
FFNN basec on features from GoogLeNet	Mild_Demented	88.8	89.12	92.51	85.5	97.68
	Moderate_Demented	69.2	69.36	85.72	90	99.73
	Non_Demented	97.7	98.29	97.25	96.9	97.18
	Very_Mild_Demented	94	93.87	94.69	95.9	98.41
	**average ratio**	**94.80**	**87.66**	**92.54**	**92.08**	**98.25**
FFNN basec on features from DenseNet-121	Mild_Demented	83.2	83.34	91.49	85.6	98.44
	Moderate_Demented	69.2	69.1	89.12	81.8	99.55
	Non_Demented	97	97.21	96.84	94.8	95.16
	Very_Mild_Demented	93.5	93.97	95.79	95.2	97.38
	**average ratio**	**93.60**	**85.91**	**93.31**	**89.35**	**97.63**

**Table 3:** Results of FFNN according to the combined features of the GoogLeNet and DenseNet-12 models.

Models	Classes of AD	Accuracy %	Sensitivity %	AUC %	Precision %	Specificity %
FFNN based on the merging of CNN features before PCA	Mild_Demented	94.4	94.27	97.52	93.9	98.96
	Moderate_Demented	69.2	69.44	84.56	69.2	99.62
	Non_Demented	98.6	98.84	98.25	98.9	99.1
	Very_Mild_Demented	97.1	97.24	96.67	96.75	98.37
	**average ratio**	**97.20**	**89.95**	**94.25**	**89.69**	**99.01**
FFNN based on the merging of CNN features after PCA	Mild_Demented	96.1	96.29	97.95	96.1	99.4
	Moderate_Demented	92.3	92.1	94.64	92.3	99.82
	Non_Demented	99.2	98.78	98.1	99.8	99.71
	Very_Mild_Demented	99.3	99.24	97.54	98.5	99.22
	**average ratio**	**98.80**	**96.60**	**97.06**	**96.68**	**99.54**

**Table 4:** FFNN performance according to the combined features between CNN models and handcrafted features.

Models	Classes of AD	Accuracy %	Sensitivity %	AUC %	Precision %	Specificity %
FFNN with features of GoogleNet and handcrafted	Mild_Demented	98.9	99.4	99.12	97.8	99.52
	Moderate_Demented	100	99.56	98.52	100	99.68
	Non_Demented	99.8	99.87	99.56	99.7	99.86
	Very_Mild_Demented	99.1	98.72	99.46	99.8	99.72
	**average ratio**	**99.50**	**99.39**	**99.17**	**99.33**	**99.70**
FFNN with features of DenseNet-121 and handcrafted	Mild_Demented	99.4	99.3	99.63	98.9	99.8
	Moderate_Demented	100	99.98	99.84	100	99.55
	Non_Demented	99.7	99.58	99.28	99.8	99.71
	Very_Mild_Demented	99.8	99.68	99.49	99.8	99.6
	**average ratio**	**999.70**	**99.64**	**99.56**	**99.63**	**99.67**

**Table 5:** Generalization of FFNN performance according to combined features between CNN models and handcrafted features on a new ADNI dataset.

Systems	Classes of ADNI Dataset	Accuracy %	Sensitivity %	AUC %	Precision %	Specificity %
FFNN with features of GoogLeNet and handcrafted	AD	88.2	88.2	89.2	85.7	97.7
	CN	98.3	98.1	93.2	95	96.4
	EMCI	87.5	87.7	93.9	95.5	98.6
	LMCI	71.4	71.2	87.6	83.3	99
	MCI	91.5	90.9	92.1	89.6	97.9
	**Average ratio**	**92.3**	**87.22**	**91.2**	**89.82**	**97.92**
FFNN with features of Dense-Net-121 and handcrafted	AD	94.1	94.2	95.1	82.1	96.9
	CN	95.7	96.1	97.2	98.2	99.2
	EMCI	95.8	95.8	96.1	95.8	98.7
	LMCI	78.6	79.4	88.4	73.3	98.1
	MCI	89.4	88.9	94.9	95.5	99.2
	**Average ratio**	**93.4**	**90.88**	**94.34**	**88.98**	**98.42**

**Table 6:** Results of all systems in this work to diagnose images of MRI for classifying of AD progression.

Techniques		Features	Mild-Dementia	Moderate-Dementia	Non-Dementia	Very-Mild-Dementia	Accuracy %
FFNN network		GoogLeNet	88.8	69.2	97.7	94	94.8
		DenseNet-121	83.2	69.2	97	93.5	93.6
FFNN network	Combined features before PCA	GoogLeNet + DenseNet-121	94.4	69.2	98.6	97.1	97.2
	Combined of features after PCA	GoogLeNet + DenseNet-121	96.1	92.3	99.2	99.3	98.8
	Combined features	GoogLeNet and handcrafted	98.9	100	99.8	99.1	99.5
		DenseNet-121 and handcrafted	99.4	100	99.7	99.8	99.7

## Data Availability

MRI image data supporting the proposed systems were obtained from a publicly available dataset at link: https://www.kaggle.com/tourist55/alzheimers-dataset-4-class-of-images (accessed on 13 September 2022).
